# ActS activates peptidoglycan amidases during outer membrane stress in *Escherichia coli*

**DOI:** 10.1111/mmi.14712

**Published:** 2021-03-23

**Authors:** Carlos K. Gurnani Serrano, Matthias Winkle, Alessandra M. Martorana, Jacob Biboy, Niccolo Morè, Patrick Moynihan, Manuel Banzhaf, Waldemar Vollmer, Alessandra Polissi

**Affiliations:** ^1^ Dipartimento di Scienze Farmacologiche e Biomolecolari Università degli Studi di Milano Milan Italy; ^2^ The Centre for Bacterial Cell Biology Biosciences Institute Newcastle University Newcastle upon Tyne UK; ^3^ Institute of Microbiology and Infection School of Biological Sciences University of Birmingham Birmingham UK; ^4^ Present address: Nikon Instruments Europe B.V Amsterdam North Holland Netherlands

**Keywords:** cell division, cell envelope, *Escherichia coli*, lipopolysaccharide, peptidoglycan

## Abstract

The integrity of the cell envelope of *E. coli* relies on the concerted activity of multi‐protein machineries that synthesize the peptidoglycan (PG) and the outer membrane (OM). Our previous work found that the depletion of lipopolysaccharide (LPS) export to the OM induces an essential PG remodeling process involving LD‐transpeptidases (LDTs), the glycosyltransferase function of PBP1B and the carboxypeptidase PBP6a. Consequently, cells with defective OM biogenesis lyse if they lack any of these PG enzymes. Here we report that the morphological defects, and lysis associated with a *ldtF* mutant with impaired LPS transport, are alleviated by the loss of the predicted OM‐anchored lipoprotein ActS (formerly YgeR). We show that ActS is an inactive member of LytM‐type peptidoglycan endopeptidases due to a degenerated catalytic domain. ActS is capable of activating all three main periplasmic peptidoglycan amidases, AmiA, AmiB, and AmiC, which were previously reported to be activated only by EnvC and/or NlpD. Our data also suggest that in vivo ActS preferentially activates AmiC and that its function is linked to cell envelope stress.

## Graphical abstract

ActS activity in stressed cells is somehow controlled by LdtF resulting in mild chaining phenotype (left panel). Deletion of *ldtF* leads to spurious ActS activation causing cell lysis (central panel). Deletion of *actS* in cells lacking LdtF suppresses the lysis phenotype (right panel). At this condition AmiC is sufficiently controlled by NlpD enabling it to partially restore cell separation.

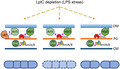

## 1 INTRODUCTION

Gram‐negative bacteria have a thin peptidoglycan (PG) layer that is surrounded by an asymmetric outer membrane (OM) which protects cells from many toxic molecules and antibiotics (Huang et al., 2008; Silhavy et al., 2010). The protective function of the OM relies on the maintenance of its asymmetric structure, with lipopolysaccharide (LPS) covering the outer surface (Henderson et al., 2016; Lundstedt et al., 2020). Growing cells transport LPS across the periplasm and through the PG layer to the OM by the Lipopolysaccharide transport (Lpt) machinery (Sperandeo et al., 2019; Whitfield & Trent, 2014). In *Escherichia coli*, the Lpt machinery has seven essential components (LptABCDEFG) that form a transient transenvelope complex from the cytoplasmic membrane (CM) to the OM (Chng et al., 2010; Okuda et al., 2016; Sperandeo et al., 2019). The Lpt machinery works as a single device and the depletion of any of its component halts LPS export, resulting in a growth arrest and the formation of short chains of unseparated cells (Ruiz et al., 2008; Sperandeo et al., 2008).

The PG layer maintains the osmotic stability and shape of a cell (Vollmer et al., 2008). In *E. coli* the majority of peptide cross‐links in PG is of 4‐3 (DD) type and formed by penicillin‐binding proteins (PBPs) (Egan et al., 2020; Typas et al., 2012), but a minority of 3‐3 (LD) cross‐links are formed by LD‐transpeptidases (LDTs) (Magnet et al., 2008). *E. coli* has six LDTs (LdtA‐F) with two distinct functions. LdtA, LdtB, and LdtC catalyze the attachment of the OM‐anchored lipoprotein Lpp to PG (Magnet et al., 2007). LdtD and LdtE form 3‐3 cross‐links (Magnet et al., 2008) whereas LdtF appears to stimulate the activity of LdtE and LdtD (Montón Silva et al., 2018; Morè et al., 2019). LDTs are not essential for growth under laboratory conditions. Single or multiple *ldt* mutants, or cells treated with sub‐MIC concentrations of copper chloride, which inhibits LDTs, display only minor phenotypes (Magnet et al., 2007; Magnet et al., 2008; Pavelka and Sanders 2013; Morè et al., 2019; Peters et al., 2018). However, LdtD, LdtE, and LdtF become essential upon OM stress, that is when the biosynthesis of LPS or its transport to the OM is compromised (Morè et al., 2019). LdtD plays a major role in survival under these conditions, but the glycosyltransferase of PBP1B, its activator LpoB, and the carboxypeptidase PBP6a are also needed, and it was proposed that these enzymes repair together defects in the PG that arise upon OM assembly stress (Morè et al., 2019).

**FIGURE 1:**
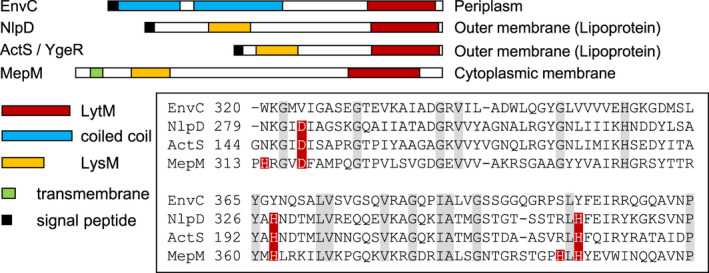
LytM domain proteins in *E. coli*. Overview of the domain organization of LytM domain proteins in *E. coli*. Sequence alignment of the LytM domain of EnvC, NlpD, ActS (YgeR), and MepM. The sequences of the LytM domains (box) show that EnvC, NlpD, and ActS have a degenerated LytM domain. Red, essential active site residues; grey, conserved residues [Colour figure can be viewed at wileyonlinelibrary.com]

During *E. coli* cell division, about 30% of newly made septal PG is removed shortly after its synthesis by hydrolases (Uehara & Park, 2008), mostly the *N*‐acetylmuramyl‐L‐alanine amidases (amidases) AmiA, AmiB, and AmiC, and lytic transglycosylases. The lack of any single amidase has a minor impact on the cell, but the loss of all three amidases results in the formation of chains of unseparated cells (Heidrich et al., 2001; Priyadarshini et al., 2007). The amidases require the activation by EnvC and NlpD, which each contains a catalytically degenerated LytM‐type endopeptidase (lysostaphin/peptidase M23) domain. AmiA and AmiB are activated by EnvC, AmiC is activated by NlpD (Peters et al., 2013; Uehara et al., 2010). LytM domains are present in many bacterial proteins, most of these function in PG hydrolysis and cell division (Meisner & Moran, 2011; Sabala et al., 2012; Zielińska et al., 2017). In addition to EnvC and NlpD, *E. coli* has two further LytM‐domain containing proteins, MepM and ActS (formerly YgeR) (Figure 1). MepM is a metallo‐endopeptidase that cleaves 4‐3 cross‐links in PG (Singh et al., 2012). ActS is an uncharacterized lipoprotein with predicted OM localization (Uehara et al., 2009). The deletion of *mepM* or *actS* slightly exacerbates the cell chaining phenotype of the double *envC nlpD* mutant (Uehara et al., 2009).

In this work, we show that ActS causes the lysis of *ldtF* mutant cells with impaired LPS transport. ActS (Amidase activator during Stress) has no PG hydrolase activity but activates all three amidases involved in septal PG, although in the cell it mainly activates AmiC through its LytM domain.

## 2 RESULTS

### 2.1 Deletion of *actS* rescues *araB*p*lptC* Δ*ldtF* cells from lysis

We showed previously that the deletion of *ldtF* (*yafK*) is lethal in cells with defective LPS transport. For example, an *araB*p*lptC* conditional mutant (with the arabinose‐inducible *araB*p promoter controlling *lptC* expression) undergoes rapid lysis if *ldtF* is deleted, similar to deletions of *ldtD* and *ldtE* (Morè et al., 2019). Notably, the *araB*p*lptC* ∆*ldtF* cells (but not *araB*p*lptC* ∆*ldtD* or *araB*p*lptC* ∆*ldtE* cells) showed abnormal size and cell shape even when grown under permissive conditions with arabinose, suggesting that LdtF might have different or additional roles compared to LdtD and LdtE (Morè et al., 2019). We therefore aimed to identify functional partners of LdtF. Searching the chemical genomics profiles of the KEIO collection of *E. coli* single mutants (Nichols et al., 2011) we noticed that the phenotypic responses of *ldtF* and *actS* mutants correlated with a high score of 0.48 across the >300 conditions tested, suggesting a functional connection between both gene products. ActS is a predicted OM‐anchored lipoprotein belonging to the LytM‐domain family of proteins (Figure 1) with an unknown function (Uehara et al., 2009). To probe for a possible link between ActS and LDTs, we combined the *actS* deletion with deletions of *ldt* genes in the background of wild‐type BW25113 (*lptC* ^+^) or the conditional *araB*p*lptC* mutant.

**FIGURE 2:**
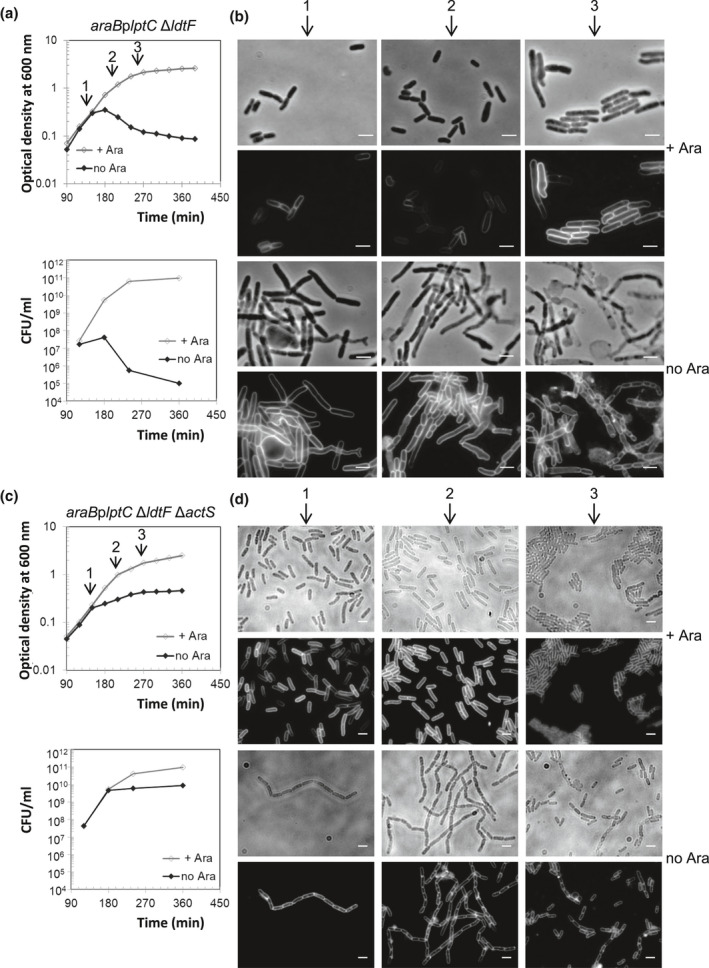
Deletion of *actS* rescues *araB*p*lptC* Δ*ldtF* cells from lysis. Cells of *araB*p*lptC* Δ*ldtF* (a) and of the isogenic mutant deleted for *actS* (c) were grown to an OD_600_ of 0.2 in the presence of 0.2% arabinose, harvested, washed three times, and resuspended in an arabinose‐supplemented (+ Ara) or arabinose‐free (no Ara) medium. Growth was monitored by OD_600_ measurements (top panel) and by determining CFU (bottom panel). Growth curves are representative of at least three independent experiments. (b and d) At *t* = 150, 210, and 270 min (arrows), cells were collected for imaging. Phase‐contrast images (top) and fluorescence images obtained by FM5‐65 staining (bottom) are shown. The deletion of *actS* also corrects morphological defects observed in *araB*p*lptC* Δ*ldtF* grown under permissive conditions. Bars, 3 μm

Indeed, we found a specific link between *actS* and *ldtF*: under nonpermissive conditions the deletion of *actS* suppressed the lysis of *araB*p*lptC* ∆*ldtF* cells, but not lysis of *araB*p*lptC* ∆*ldtD* or *araB*p*lptC* ∆*ldtE* cells (Figure 2, Figure S1). Phase‐contrast and fluorescence microscopy of *araB*p*lptC* Δ*ldtF* Δ*actS* cells further showed that the deletion of *actS* also corrected the morphological defects of a*raB*p*lptC* Δ*ldtF* cells under permissive condition (Morè et al., 2019) (Figure 2). Notably, during early depletion *araB*p*lptC* Δ*ldtF* mutant displays many cells with no septa, whereas *araB*p*lptC* Δ*ldtF* Δ*actS* mutant forms chains of cells in early depletion and more single cells in late depletion. Growth and cell morphology were normal in *lptC* ^+^ strains when *actS* was deleted alone or in combination with *ldtD*, *ldtE*, or *ldtF* (Figures S2 and S3), indicating that the observed defects are specific to OM‐stressed cells.

**FIGURE 3:**
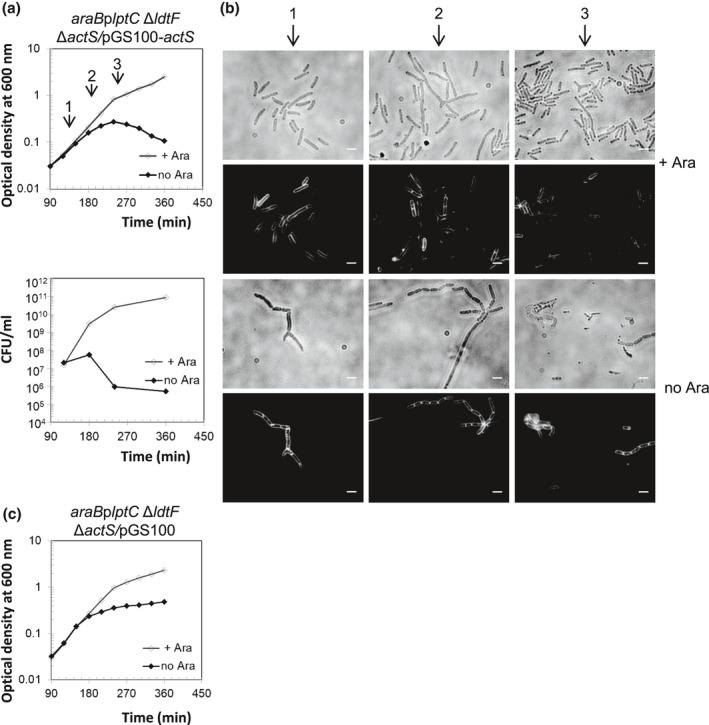
Ectopic expression of *actS* restores lysis phenotype in *araB*p*lptC* Δ*ldtF* Δ*actS*. Cells of *araB*p*lptC* Δ*ldtF* Δ*actS* strains carrying pGS100‐*actS* (a) were grown in the presence of 0.2% arabinose and Cam to an OD_600_ of 0.2, harvested, washed three times, and resuspended in an arabinose‐supplemented (+ Ara) or arabinose‐free (no Ara) medium. The p*tac* promoter in pGS100‐*actS* is leaky, expressing downstream genes even in the absence of the inducer IPTG. Cell growth was monitored by OD_600_ measurements (top panel) and by determining CFU (bottom panel). Growth curves are representative of at least three independent experiments. (b) At *t* = 180, 240, and 300 min (arrows), cells were collected for imaging. Phase‐contrast images (top) and fluorescence images obtained by FM5‐65 staining (bottom) are shown. Bars, 3 μm. Growth of *araB*p*lptC* Δ*ldtF* Δ*actS* cells expressing pGS100 void plasmid is shown as control (c)

We further confirmed that *araB*p*lptC* Δ*ldtF* Δ*actS* cells complemented with an ectopic copy of *actS* expressed from pGS100‐*actS* undergo lysis (Figure 3). The *araBplptC* cells expressing *actS* do not lyse under nonpermissive conditions but display cell separation defects (branching cells and cells with no septa) (Figure S4). This result strongly suggests that the absence of *actS* is the reason for lysis suppression in *araB*p*lptC* Δ*ldtF* Δ*actS* cells and established that ActS causes the lysis of *araB*p*lptC* Δ*ldtF* cells under nonpermissive condition.

### 2.2 Lysis protection by ActS removal does not enhance the degree of 3‐3 cross‐links

The lysis phenotype of the *araB*p*lptC* Δ*ldtF* strain is also suppressed by the deletion of *ldtE*. In this strain, the expression of LdtD, the major stress‐response LDT in *E. coli*, is strongly induced, resulting in increased levels of 3‐3 cross‐links in the PG which protect cells from lysis (Morè et al., 2019). We therefore analyzed the PG composition of the *araB*p*lptC* Δ*ldtF* Δ*actS* strain to assess whether suppression of the lysis phenotype correlates with an increased level of 3‐3 cross‐links. We found that the level of 3‐3 cross‐links in the *araB*p*lptC* Δ*ldtF* Δ*actS* mutant was comparable to that of the *araB*p*lptC* strain and its derivative deleted for *actS* under permissive and nonpermissive conditions (Table 1 and Table S1). Because the lack of ActS has no effect on the level of 3‐3 cross‐links, we concluded that lysis protection in the *lptC*‐depleted *ldtF* mutant cells does not require the strengthening of the PG layer by introducing additional crosslinks.

**TABLE 1:** Summary of the level of 3‐3 cross‐links in PG and growth phenotype of *ldt* mutant strains, combined with *actS* deletion, in *lptC* depleted or not depleted cells

Gene				3‐3 Cross‐linkage/phenotype				
				*lptC* ^+^	*araB*p*lptC*			
					+ Arabinose		No arabinose	
*ldtF*	*ldtD*	*ldtE*	*actS*	Growth	Growth	3‐3 Cl (area %)^a^	Growth	3‐3 Cl (area %)^a^
+	+	+	+	Normal	Normal	2.0	Arrest	6.7
+	+	+	−	Normal	Normal	2.5	Arrest	6.3
+	−	+	−	Normal	Normal	NT	Lysis	NT
+	+	−	−	Normal	Normal	NT	Lysis	NT
−	+	+	−	Normal	Normal	2.2	Arrest	7.4
−	+	+	+	Normal^b^	Normal^b^	1.9^b^	Lysis^b^	8.4^b^
−	+	−	+	Normal^b^	Normal^b^	8.2^b^	Arrest^b^	8.4^b^

Abbreviations: CL, cross‐links; NT, not tested.

^a^ Sum of the percentages of all muropeptides with 3‐3 cross‐links (CL) in the muropeptide profile. See Table S1 for complete data on the muropeptide composition.

^b^ Data from Morè et al. (2019).

### 2.3 ActS has a degenerate LytM domain and does not cleave PG

**FIGURE 4:**
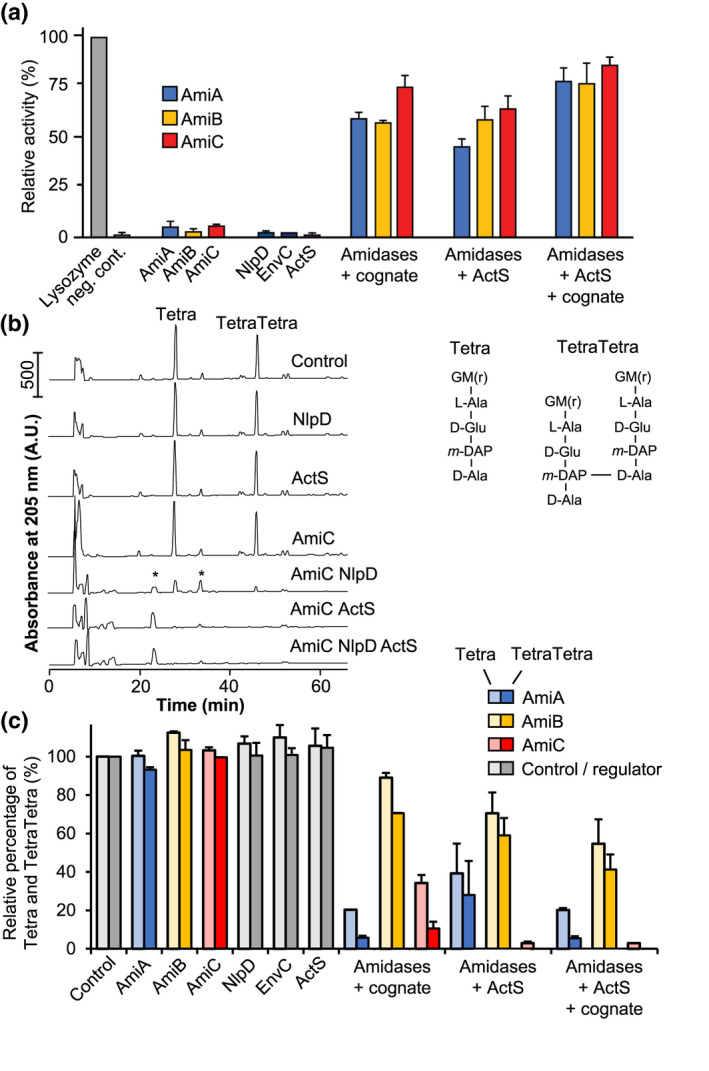
PG degradation assay representing amidase activity. (a) The graph shows the relative degradation of FITC labeled sacculi by amidases and/or regulators compared with a lysozyme control. ActS activates all tested amidases in a similar fashion to their previously known cognate regulators (EnvC for AmiA/B, NlpD for AmiC). Adding ActS and NlpD or ActS and EnvC results in an increase in PG degradation. The data shown in this graph represents two biological replicates. (b) Example HPLC chromatograms showing the activation of AmiC by its cognate regulator NlpD or ActS. PG sacculi were incubated with the proteins indicated, and the reaction was terminated by boiling for 10 min. Muropeptides were released by cellosyl and separated by HPLC. Asterisks indicate peptide products of amidase activity, the glycans are not visible. Hence, amidase activity is best followed by the reduction in Tetra and TetraTetra muropeptide. Example HPLC chromatograms of AmiA and AmiB digests are shown in Figure S5 G, *N*‐acetylglucosamine; *M*(r), *N*‐acetylmuramitol; L‐Ala, L‐alanine; D‐Glu, D‐glutamic acid; D‐Ala, D‐alanine; *m*‐DAP, *meso*‐diaminopimelic acid. (c) Quantification of Tetra and TetraTetra remaining at the end of the reaction with the proteins indicated as a measure of amidase activity, quantified by HPLC analysis as in (b). Values are mean ± variation of two independent samples [Colour figure can be viewed at wileyonlinelibrary.com]

ActS has a predicted N‐terminal LysM (Lysin Motif) PG‐binding domain (Buist et al., 2008) and a C‐terminal catalytic LytM (lysostaphin/M23 peptidase) domain (Peters et al., 2013; Tsang et al., 2017). Essential catalytic residues of LytM from *Staphylococcus aureus* (Peters et al., 2013) are conserved in the PG endopeptidase MepM but not in the catalytically inactive amidase activators EnvC and NlpD (Figure 1) (Singh et al., 2012; Uehara et al., 2010). The LytM domain of ActS shares 65%, 35%, and 34% sequence identity with the LytM domains of NlpD, EnvC, and MepM, respectively (Figure 1). Importantly, out of the five essential catalytic residues in MepM (H314, D318, H362, H393, and H395), only three are conserved in ActS (D149, H194, and H226) (Figure 1). We therefore hypothesized that ActS has a degenerated LytM domain‐like EnvC and NlpD. To test this hypothesis, we purified ActS and tested its possible endopeptidase activity in two ways, against fluorescent labeled or unlabeled PG. We incubated fluorescent labeled PG with ActS, and then measured the fluorescence in the supernatant to detect any possible fluorescent PG fragments released (Figure 4a). We also incubated unlabeled PG with ActS, followed by digestion with the muramidase cellosyl and the separation of the resulting muropeptides by high‐performance liquid chromatography (HPLC) (Figure 4b,c). Both experiments showed that ActS does not have PG hydrolase or endopeptidase activity, consistent with the degenerated active site (Figure 4 and Figure S5).

### 2.4 ActS activates three PG amidases

We next considered that, like EnvC and NlpD, ActS might function as an amidase activator. We tested this hypothesis by incubating purified AmiA, AmiB, or AmiC with or without ActS, using FITC‐stained PG sacculi as substrate to measure the release of soluble FITC‐labeled amidase products (Figure 4a). EnvC or NlpD were also tested with their cognate amidase(s) in the presence or absence of ActS. The amidases, activators, or ActS alone released hardly any PG fragments whilst the amidases were active in the presence of their cognate activator, EnvC or NlpD. Interestingly, the addition of ActS activated all three amidases, and the combination of ActS with a cognate activator further increased amidase activity.

To verify amidase activity in the presence of ActS, we digested the reaction products from unlabeled PG with cellosyl and analyzed the resulting PG fragments by HPLC (Figure 4b,c and Figure S5). These data show that ActS activated AmiA, AmiB, and AmiC to digest PG, best seen as the disappearance of the canonical monomeric and dimeric muropeptides (Tetra and TetraTetra, respectively). In this assay, we noticed that ActS‐activated AmiC digested more Tetra and TetraTetra substrates than NlpD‐activated AmiC (Figure 4b,c). Together these results indicate that ActS is capable of activating AmiA, AmiB, and AmiC, which is distinct to the more specific activators EnvC and NlpD (Uehara et al., 2010).

### 2.5 ActS interacts with AmiC and binds PG

**FIGURE 5:**
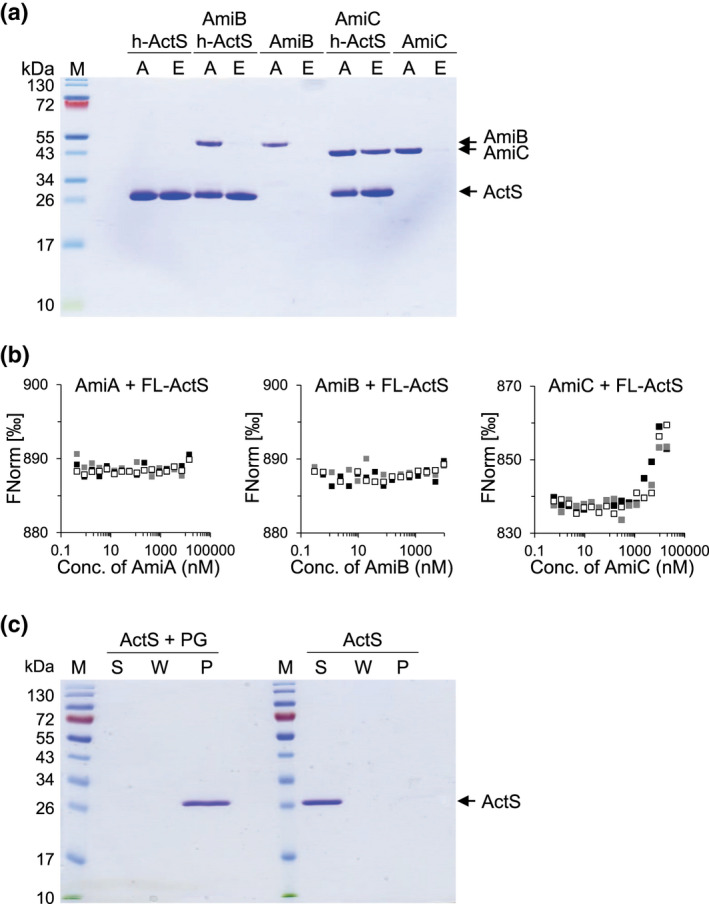
ActS interacts with AmiC and binds PG. A, AmiC is pulled down by His‐ActS. Coomassie blue‐stained SDS–PAGE gel showing the pull‐down of proteins to Ni^2+^‐NTA beads. (a) applied sample; E, eluted sample. (b) Fluorescent (NT‐647)‐labeled ActS (FL‐ActS) interacts with AmiC, but no AmiA or AmiB, in microscale thermophoresis (MST). FL‐ActS (100 nM) was titrated against two‐fold serial dilutions of unlabeled AmiA, AmiB, or AmiC, and the thermophoresis (movement along a temperature gradient) of FL‐ActS was followed and quantified as normalized fluorescence (Fnorm). A change in Fnorm indicates ligand‐binding. (c) ActS is pulled down by PG. Coomassie blue‐stained SDS–PAGE gel showing the pull‐down of ActS to PG from strain BW25113Δ6LDT. ActS was present in the pellet fraction (lane P) in samples with PG but not without PG. S, supernatant; W, wash [Colour figure can be viewed at wileyonlinelibrary.com]

We then assayed whether ActS physically interacts in vitro with amidases by mixing oligohistidine‐tagged ActS with untagged AmiA, AmiB, or AmiC and pull‐down to Ni^2+^‐NTA agarose beads. AmiC was pulled down by oligohistidine‐tagged ActS (Figure 5a), indicating a direct interaction between both proteins. We did not observe the pull‐down of AmiB with oligohistidine‐tagged ActS (Figure 5a) and could not analyze AmiA because of the similar molecular weight of AmiA and ActS precluded their separation by SDS–PAGE (not shown). We also attempted to detect the interaction between fluorescence‐labeled ActS (FL‐ActS) and amidases by microscale thermophoresis (MST). Only AmiC at high concentration caused a change thermophoresis of FL‐ActS (Figure 5b), suggesting that possible interactions between ActS and amidases are weak and/or transient and again pointing to a stronger effect toward AmiC.

ActS has a LysM domain and these are predicted to bind PG (Buist et al., 2008; Mesnage et al., 2014). We therefore performed an in vitro PG pull‐down assay with ActS which showed that ActS was indeed present in the PG fraction, confirming its ability to bind PG (Figure 5c).

### 2.6 Expression of ActS restores cell separation in the *nlpD envC* double mutant

**FIGURE 6:**
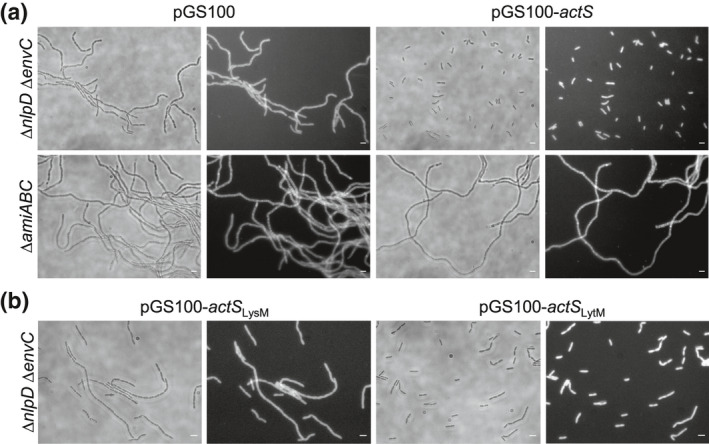
ActS overproduction restores cell separation in amidase activator‐deficient mutants. (a) Representative images of BW25113 ∆*nlpD* ∆*envC* and BW25113 ∆*amiABC* cells overexpressing full length *actS* (pGS100‐*actS*). (b) Representative images of overexpression of *actS lytM* (pGS100‐*actS*_LytM_) or *lysM* (pGS100‐*actS*_LysM_) domains BW25113 ∆*nlpD* ∆*envC*. Images of BW25113 ∆*nlpD* ∆*envC* and BW25113 ∆*amiABC* harboring an empty plasmid (pGS100) are also shown as control. Cells were grown overnight and collected for imaging. Phase‐contrast images (left) and fluorescence images obtained by FM5‐65 staining (right) are shown. Size bars, 3 μm

Our biochemical data showed that ActS is a novel amidase activator; however, the Δ*nlpD* Δ*envC* double mutant exhibits extensive cell chaining due to the lack of activation of AmiA, AmiB, and AmiC (Uehara et al., 2010), despite the presence of *actS*. This suggests that the level of *actS* in the Δ*nlpD* Δ*envC* mutant is not sufficient to activate the amidases. To test this hypothesis, we expressed *actS* from plasmid pGS100‐*actS* in the Δ*nlpD* Δ*envC* mutant and assessed the cell chaining phenotype. The p*tac* promoter in pGS100 is leaky, expressing downstream genes even in the absence of the inducer IPTG. Cells were grown in the absence of IPTG and collected for imaging. As shown in Figure 6, the ectopic expression of *actS* restored cell separation in the Δ*nlpD* Δ*envC* mutant but not in the triple amidase (Δ*amiABC*) mutant used as a control, thus confirming that ActS functions as an amidase activator in cells when expressed at sufficient level. This is in line with low ActS abundance under nonstress conditions as assessed by ribosome profiling (Oh et al., 2011).

The LytM domain in NlpD or EnvC is critical for amidase activation (Peters et al., 2013; Tsang et al., 2017; Uehara et al., 2010). Hence, we next asked whether the LytM domain in ActS is responsible for amidase activation. We generated expression plasmids for the LysM (pGS100‐*actS*_LysM_) or LytM (pGS100‐*actS*_LytM_) domain and introduced these into the Δ*nlpD* Δ*envC* strain. The LytM domain of ActS largely restored cell separation (Figure 6b), although a mild chaining phenotype was still observed. On the contrary, Δ*nlpD* Δ*envC* cells expressing the LysM PG‐binding domain still display the chaining phenotype. These results show that the LytM domain is required for amidase activation whereas the LysM domain is not required but increases the efficiency.

### 2.7 ActS preferentially activates AmiC in vivo

**FIGURE 7:**
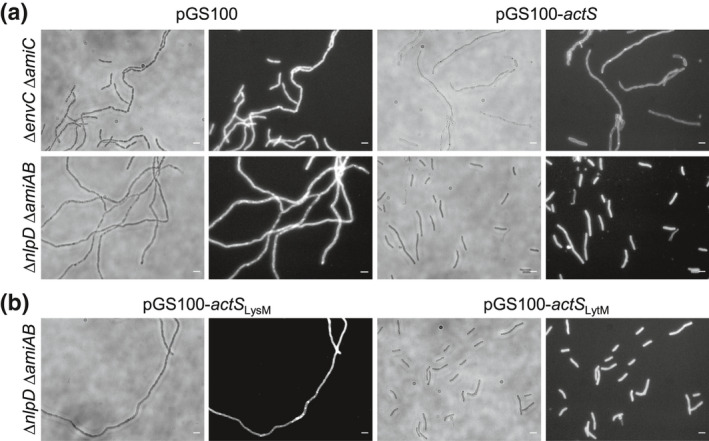
ActS activates preferentially AmiC for cell separation. Representative images of BW25113 ∆*envC* ∆*amiC* or BW25113 *∆nlpD ∆amiAB* cells expressing full length *actS* (pGS100‐*actS*) (a). Representative images of BW25113 *∆nlpD ∆amiAB* cells expressing *actS* LytM (pGS100‐*actS*_LytM_) or LysM (pGS100‐*actS*_LysM_) domains (b). Images of BW25113 ∆*envC* ∆*amiC* and BW25113 *∆nlpD ∆amiAB* harboring an empty plasmid (pGS100) are also shown as control. Cells were grown overnight and collected for imaging. Phase‐contrast images (left) and fluorescence images obtained by FM5‐65 staining (right) are shown. Size bars, 3 μm

Our data so far support the hypothesis that ActS is an additional activator of AmiA, AmiB, and AmiC. The amidases have redundant roles in septum PG cleavage and cell separation (Priyadarshini et al., 2006). ActS interacts with AmiC (Figure 5a,b) and activates AmiC better than NlpD (Figure 4b,c), and the LytM domain of ActS has the highest sequence similarity to that of NlpD (Figure 1), suggesting that ActS might have a preference for AmiC in the cell. To test this hypothesis, the pGS100‐*actS* plasmid was introduced into the Δ*envC* Δ*amiC* and Δ*nlpD* Δ*amiAB* mutants, and the chaining phenotype was assessed. The ectopic expression of ActS alleviated the cell separation defects in Δ*nlpD* Δ*amiAB*, but not Δ*envC* Δ*amiC* cells (Figure 7), suggesting that ActS preferentially activates AmiC.

We next assessed whether the lysis phenotype of the *araB*p*lptC* Δ*actS* mutant can be rescued by the deletion of *amiC* and found that lack of AmiC does not protect from lysis under nonpermissive conditions (Figure S6a). This result is consistent with data reported in the accompanying manuscript (Mueller et al., 2021) where AmiA is shown to be sufficient for normal cell separation under neutral and acidic conditions. Finally, we show that deletion of *nlpD* does not suppress the lysis phenotype of *lptC*‐depleted *actS*‐deleted cells (Figure S6b), further supporting the notion that there is a specific functional connection between LdtF and ActS.

## 3 DISCUSSION

Bacterial cells must preserve the integrity of their sacculus at all times and under a variety of growth and stress conditions to prevent lysis and death (Egan et al., 2020). Here we studied cell survival upon OM biogenesis stress due to depletion of *lptC*, which led us to identify a novel activator of PG amidases, the OM‐anchored lipoprotein ActS. Of all PG hydrolases, amidases have the highest contribution to septum cleavage during cell division (Heidrich et al., 2001; Priyadarshini et al., ,^2006^, ^2007^). ActS possesses a degenerate LytM domain and does not cleave PG, similar to the amidase activators EnvC and NlpD (Uehara et al., 2010), (Figure S5), and ActS has a LysM domain and binds PG as does NlpD (Figure 5c). Unlike EnvC and NlpD, ActS activates the three amidases, AmiA, AmiB, and AmiC in vitro (Figure 4a and Figure S5). These biochemical data are consistent with our cellular data showing that the ectopic expression of *actS* fully restored cell separation in a double‐mutant deficient for *nlpD* and *envC* (Figure 6). Our cellular data also suggest a possible preference of ActS for AmiC, which is consistent with the similarities between ActS and the other AmiC activator, NlpD. Both activators are lipoproteins anchored to the outer membrane, have a LysM PG‐binding domain, and a degenerated LytM domain for AmiC activation.

Previously it was thought that each amidase has a dedicated activator (Uehara et al., 2010). Here we showed that AmiC is not only activated by the previously identified NlpD but is also by ActS. In the accompanying paper, the Levin group shows that cells growing at acidic conditions rely mostly on AmiB and its activation by NlpD, EnvC, and ActS for cell separation (Mueller et al., 2021). Hence, the simplistic view of each amidase having one activator has to be revised and more work is needed to decipher the mechanisms of PG cleavage during cell division under different growth conditions.

**FIGURE 8:**
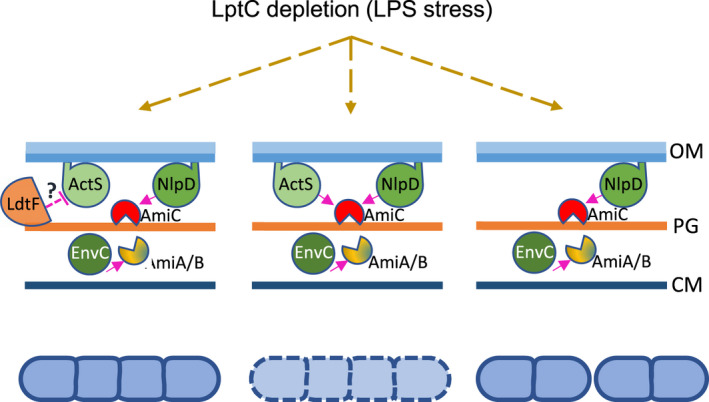
Model for ActS activation upon LPS stress. Upon block of LPS transport LdtF indirectly affects the ActS‐mediated activation of amidases (dashed line) resulting in a mild chaining phenotype (left panel). Without LdtF ActS spuriously activates amidases causing cell lysis (central panel). Deletion of *actS* in in cells lacking LdtF suppresses the lysis phenotype (right panel). At this condition AmiC is sufficiently controlled by NlpD enabling it to partially restore cell separation [Colour figure can be viewed at wileyonlinelibrary.com]

In the case of EnvC, the tight spatio‐temporal control of amidase activation is achieved by the auto‐inhibition of the activator which is relieved by FtsEX, thus coordinating periplasmic peptidoglycan remodeling with cytoplasmic cell division events (Yang et al., 2011; Cook et al., 2020). YraP (DolP) and the Tol‐Pal system are implicated in the activation of NlpD to coordinate OM constriction and PG remodeling during cytokinesis (Tsang et al., 2017). Our data suggest that the ability of ActS to modulate AmiC activity is somehow controlled by LdtF (Figure 8) which could be caused by an altered PG architecture in stressed *ldtF* deleted cells and not by a direct interaction between the two proteins. This hypothesis is supported by our recent discovery that LdtF (now DpaA) detaches Lpp from the PG (Winkle et al., 2021). Overall, these data add further complexity to the amidase regulation network.

ActS function appears to be relevant under envelope stress conditions and the protein seems to be poorly expressed or not functional in unstressed cells, explaining why the Δ*nlpD* Δ*envC* double‐mutant displays a severe cell chaining phenotype despite carrying a functional copy of *actS*. Based on the EcoCyc database (Keseler et al., 2011; http://ecocyc.org/), *actS* resides in a monocistronic operon that is preceded by a putative σ^E^‐dependent promoter consensus sequence 186 nucleotides upstream of the *actS* TTG start codon. σ^E^‐responds to extra‐cytoplasmic stress and activates, for example, genes that counteract cell envelope damage due to LPS transport defects (Klein et al., 2016; Martorana et al., 2011, 2014). This is consistent with our observations that the overexpression of *actS* in *lptC*‐depleted cells or the deletion of *actS* in *lptC*‐depleted *ldtF*‐deleted cells cause cell separation defects. It is known that cell chaining due to the lack of amidase activity leads to enhanced OM permeability making the cell chains susceptible to sodium dodecyl sulphate or vancomycin (Heidrich et al., 2002). Others have previously hypothesized that AmiC may be cross‐activated by other LytM factors to promote daughter cell separation and maintain cell envelope integrity (Tsang et al., 2017). Hence, the stress‐induced expression of ActS in mildly chaining *lptC*‐depleted cells likely functions to activate amidases to alleviate OM problems associated with the cell chaining phenotype.

How does LdtF prevent cell lysis under these conditions? We speculate that the lack of LdtF, which causes Lpp to be permanently attached to PG thus impacting on the cell's ability to remodel the PG and affecting cell envelope architecture (Winkle et al., 2021), triggers an overactivation of AmiC by ActS, causing morphological defects even under permissive conditions and lysis upon *lptC* depletion (Morè et al., 2019). Therefore, the deletion of *actS* in this mutant would suppress the detrimental effects produced by AmiC overactivation (Figure 8). Further work is needed to dissect at the molecular level the mechanisms that lead ActS to activate AmiC in the absence of LdtF. Overall, our work highlights the intricate connections between PG remodeling and amidase activation in response to impaired OM biogenesis, which together ensure robust maintenance of cell envelope integrity under stress.

## 4 EXPERIMENTAL PROCEDURES

### 4.1 Bacterial strains, plasmids, and growth conditions

*Escherichia coli* strains and plasmids used in this work are listed in Tables S2 and S3, respectively. Oligonucleotides are listed in Table S4. Cells were routinely grown aerobically at 37°C or 30°C in LB‐Lennox medium (10 g/L tryptone, 5 g/L yeast extract, 5 g/L NaCl) (Difco). When required, antibiotics or inducers were added: ampicillin (100 g/ml, Amp), chloramphenicol (25 µg/ml, Cam), kanamycin (25 µg/ml, Kan), arabinose (0.2% [wt/vol], Ara). For LptC depletion, bacteria were harvested from cultures with an OD_600_ of 0.2 by centrifugation, washed twice with LB‐Lennox, and diluted 100‐fold in LB‐Lennox with or without arabinose. Cell growth was monitored by OD_600_ measurements, and viability was determined by quantifying the colony‐forming units (CFU).

### 4.2 Construction of *E. coli* deletion or depletion strains

Deletion strains were obtained by moving *kan*‐marked alleles from the Keio *E. coli* single‐gene knockout library (Baba et al., 2006) by P1 phage transduction (Silhavy et al., 1984) or by one‐step inactivation of chromosomal genes as described in Datsenko and Wanner (2000). The *kan* cassette was removed by pCP20‐encoded Flp recombinase to generate unmarked deletions with an FRT‐site scar sequence (Datsenko & Wanner, 2000). The removal of the *kan* gene was verified by PCR. Strains with multiple deletions were generated by sequential P1 transduction and *kan* cassette removal. LptC depletion strains were obtained by moving the *kan araC araB*p‐*lptC* allele from BB‐3 (Sperandeo et al., 2006) into selected mutants by P1 transduction. Depletion strains were selected on media containing kanamycin and 0.2% arabinose. The insertion of the cassette was verified by PCR.

### 4.3 Construction of plasmids

pGS100‐*actS* was constructed by cloning *actS* between the *EcoR*I/*Hind*III restriction sites of the plasmid pGS100 (Sperandeo et al., 2006), using the primers AP618/AP619. pGS100‐*actS*_LysM_ was constructed by cloning *actS* from the TTG start codon to the position 318, using the primers AP618/AP703 into *EcoR*I/*Hind*III restriction sites of pGS100. pGS100‐*actS*_LytM_ was constructed by two‐step PCR as follow. Briefly, two fragments flaking the *actS* signal sequence were PCR amplified using pGS100‐*actS* as template and the primer pairs AP618/AP704 to generate fragment SS (encoding the signal sequence), and AP705/AP619 to generate fragment LytM (encoding the LytM domain sequence). Fragments SS and LytM were used as template for a second round of PCR amplification using AP618 and AP619 primers. The resulting amplification product was cloned into EcoRI/HindIII restriction sites of pGS100. The correct nucleotide sequences of inserts were verified by sequencing (Eurofins Genomics).

For pET28a‐His‐ActS, *actS* was cloned starting from position 129 downstream the TTG start codon, into NdeI/XhoI pET28a, using AP677 and AP621 primers. The correct nucleotide sequences of inserts were verified by sequencing (Eurofins Genomics).

For pET28a‐His‐EnvC, *envC* was cloned into a pET28a plasmid using *envC*‐f and *envC*‐r as forward and reverse primers, respectively.

### 4.4 Imaging and image analysis

Cells grown overnight were collected to obtain a total amount corresponding to an OD_600_ of 3, and a 1:10 ratio of fixation solution (fixation solution: formaldehyde 37%–glutaraldehyde 25% in PBS) was added. Cells were incubated for 30 min at 37°C, with shaking, washed with PBS, and resuspended in 0.5 ml of PBS. A cell suspension (5 μl) was spotted onto a microscope slide coated with a thin layer of 1% agarose. Images were acquired with a Zeiss Axiovert 200 M microscope coupled with an AxioCam Mrm device camera (Zeiss) and with Metamorph imaging software (Universal Imaging). For membrane staining, cells were mounted on a slide coated with 1% agarose supplemented with the membrane dye FM5‐95 (ThermoFisher) to a final concentration of 2 μg/ml (Morè et al., 2019). Images were analyzed with ImageJ (http://rsb.info.nih.gov/ij/).

### 4.5 Purification of ActS

*E. coli* LOBSTR‐BL21(DE3) (Kerafast) cells carrying the plasmid pET28‐His‐ActS were grown in 1 L of LB medium at 37°C until an OD_600_ of 0.5 was reached. IPTG (1 mM) was added and the cells were grown for 3 hr, chilled on ice for 15 min, harvested by centrifugation for 15 min at 6,000× *g*, and incubated at 4⁰C. The cell pellet was resuspended in 60 ml of Buffer I (20 mM Tris–HCl pH 7.5, 1 M NaCl, 10 mM MgCl_2_, 10% glycerol) supplemented with protease inhibitor cocktail (Sigma‐Aldrich; 1/1,000 dilution), small amount of DNase and 100 μM phenylmethyl sulfonyl fluoride (Sigma Aldrich). Cells were broken by sonication, and the soluble fraction was removed after ultracentrifugation for 1 hr at 130,000 g and 4°C. The supernatant was recovered, mixed with 2.5 ml of Ni^2+^‐NTA superflow beads (Qiagen) preequilibrated in Buffer II (20 mM Tris–HCl pH 7.5, 500 mM NaCl, 10 mM MgCl_2_, 10% glycerol) and incubated for 3 hr at 4°C with mixing. The suspension was poured in a gravity flow column and washed with 25 ml of Buffer II (supplemented with 30 mM imidazole). His‐ActS was eluted with Buffer III (20 mM Tris–HCl pH 7.5, 500 mM NaCl, 10 mM MgCl_2_, 10% glycerol, 400 mM imidazole). Elution fractions containing His‐ActS were pooled together and dialyzed against 2 L dialyze buffer (20 mM HEPES–NaOH pH 7.5, 300 mM NaCl, 10 mM MgCl_2_, 10% glycerol) overnight. Dialyzed proteins were concentrated and further purified by size exclusion chromatography on a HiLoad 16/600 Superdex 200 pg (GE Healthcare) column using size exclusion buffer (20 mM HEPES/ NaOH pH 7.5, 300 mM NaCl, 10 mM MgCl_2_, 10% glycerol) and at a flowrate of 1 ml/min. Purity was determined by SDS–PAGE, and combined fractions were concentrated and stored in aliquots at −80°C. For the removal of the His‐tag, thrombin (1.32 U/μl, Novagen) was added, and the sample was dialyzed in 3 × 1 L of cleavage buffer (20 mM HEPES/NaOH pH 7.5, 300 mM NaCl, 10% glycerol).

### 4.6 Purification of *E. coli* amidases and cognate regulators

The *E. coli* amidases AmiA, AmiB, AmiC, and their cognate regulators NlpD and EnvC/ EnvC_LytM_ were purified as described previously (Uehara et al., 2010).

### 4.7 In vitro pull‐down assays

In vitro pull‐down assays were performed as described in Gray et al., 2015 without the addition of the cross‐linker formaldehyde to the samples. Proteins (2 μM) were mixed in 200 μl of binding buffer (10 mM HEPES/NaOH pH 7.5, 10 mM MgCl_2_, 150 mM NaCl, 0.05% Triton X‐100) Samples were incubated for 30 min on ice followed by 10 min at room temperature. For the pull‐down approach, 250 µl binding buffer and 50 µl of washed and equilibrated Ni^2+^‐NTA superflow beads (Qiagen) were added to the samples (final volume of 500 µl) and incubated for 3 hr at 4°C, with mixing. Beads were washed with 6 × 500 µl wash buffer (10 mM HEPES/NaOH pH 7.5, 10 mM MgCl_2_, 500 mM NaCl, 40 mM imidazole, 0.05% Triton X‐100). Bound proteins were recovered by boiling the beads in SDS–PAGE loading buffer. Beads were removed by centrifugation and proteins resolved by SDS–PAGE. Gels were stained with Coomassie Brilliant Blue.

### 4.8 Microscale thermophoresis (MST)

MST was performed as described in Morè et al., (2019), using 100 nM fluorescent labeled ActS (FL‐ActS) and unlabeled amidases in the final buffer condition 50 mM Tris–HCl pH 8, 150 mM NaCl, 5% glycerol, 0.025% TX‐100.

### 4.9 In vitro peptidoglycan binding assay

The in vitro binding assay was performed as previously described (Ursinus et al., 2004). Purified ActS (30 μg) was incubated for 30 min at 4°C either with or without PG (purified from BW25113Δ6LDT) in binding buffer (10 mM Tris–maleate pH 6.8, 10 mM MgCl_2_, 50 mM NaCl) in a total volume of 100 μl. The samples were centrifuged for 20 min at 13,000 *g* (4°C). The resulting pellets were washed in 200 μl of binding buffer, resuspended in 100 μl of 2% SDS, and incubated for 1 hr at 4°C with mixing. Supernatants of the binding step, washing steps, and the resuspended pellets were analyzed by SDS–PAGE.

### 4.10 PG sacculi preparation

The PG sacculi was prepared as previously described (Glauner et al., 1988). *E. coli araB*p*lptC* conditional strains were grown, collected, and processed to obtain sacculi samples as described in Morè et al., (2019).

### 4.11 HPLC analysis of muropeptides

HPLC analysis was carried out as previously described (Glauner et al., 1988). PG sacculi (100 μl) were digested with cellosyl (1 μM) at 37°C overnight (Hoechst). Muropeptide samples were reduced with sodium borohydride as described (Glauner et al., 1988). Muropeptides were separated by reversed‐phase HPLC in a 180‐min linear gradient from 50 mM NaPO_4_ pH 4.31 to 75 mM NaPO_4_ pH 4.95, 15% methanol. Muropeptides were detected by absorbance at 205 nm. The software Laura (LabLogic) was used for data acquisition and analysis.

### 4.12 Preparation of sacculi labeled with FITC

PG sacculi were isolated from *E. coli* Top10 as described previously (Glauner et al., 1988). The purified sacculi were subsequently labeled with fluorescein isothiocyanate (FITC) as described in (Moynihan et al., 2019). Briefly, 25 mg of PG was incubated with 12.5 mg of FITC in 3 ml 0.5 M sodium bicarbonate buffer pH 9.3. This was incubated at 37°C in the dark for 4 hr and unreacted FITC was washed away with three volumes of buffer and washed with water until residual FITC could no longer be detected. It was then washed with two volumes of acetone, dried, and stored in the dark.

### 4.13 In vitro PG degradation assay using FITC labeled sacculi

To probe in vitro activity, purified amidases and their regulators were mixed with FITC labeled PG sacculi (FITC‐PG) to assay PG degradation. Hereby, 10 μl of a FITC‐PG suspension was incubated at 37°C for 1 hr with 1 μΜ purified amidases and/or regulators in 100 μl of reaction buffer (50 mM Tris–HCl pH 8.0, 150 mM NaCl). As a positive control, 4 μΜ lysozyme and no protein was added to the same amount of sacculi and reaction buffer. After 1 hr, the reaction was stopped by filtration and the fluorescence of the soluble fraction was read (Ex. 495, Em, 519) in a BMG Fluorostar plate reader.

### 4.14 In vitro ActS activity assays

PG digestion assays and subsequent muropeptide composition analysis by HPLC were carried out as previously described (Glauner, 1988). Ten μl (∼150 µg) of isolated PG from *E. coli* MC1061 was incubated with of purified AmiA, AmiB, AmiC, NlpD, and/or EnvC_LytM_ (2 μM) in the presence of 20 mM HEPES/NaOH pH 7.5, 100 mM NaCl, 1 mM ZnCl_2_, 0.05% Triton X‐100, in a total volume of 50 μl at 37°C for 1 hr. Reactions were terminated by boiling for 10 min at 100°C, and the remaining PG was digested overnight at 37°C with the muramidase cellosyl (1 μM). Samples were centrifuged at 10,000 *g* for 5 min, and the muropeptides were recovered from the supernatant. Muropeptides were reduced with sodium borohydride and analyzed by reversed‐phase HPLC (Glauner, 1988).

## Supporting information

Fig S1‐S6‐Table S2‐S4

Table S1
