# Supplementary material for: ActS activates peptidoglycan amidases during outer membrane stress in Escherichia coli
Source: Mol Microbiol. 2021 Mar 23;116(1):329–42. doi: 10.1111/mmi.14712 (PMC8360153; doi:10.1111/mmi.14712)
Supplement: Supplementary file 1 — Fig S1‐S6‐Table S2‐S4 [file MMI-116-329-s002.pdf]

SUPPORTING INFORMATION

ActS activates peptidoglycan amidases during outer membrane stress in *Escherichia coli*

Carlos K. Gurnani Serrano[1], Matthias Winkle[2], Alessandra M. Martorana[1], Jacob Biboy[2], Niccolo Morè[1§], Patrick Moynihan[3], Manuel Banzhaf[3], Waldemar Vollmer[2#] and Alessandra Polissi[1#]

[1] Dipartimento di Scienze Farmacologiche e Biomolecolari, Università degli Studi di Milano, Milan, Italy.

[2] The Centre for Bacterial Cell Biology, Biosciences Institute, Newcastle University, Newcastle upon Tyne, NE3 2AX, United Kingdom.

[3] Institute of Microbiology and Infection, School of Biological Sciences, University of Birmingham, Birmingham, B15 2TT, United Kingdom.

[§] Present address: Nikon Instruments Europe B.V., Amsterdam, North Holland, Netherlands.

[#]Address correspondence to Alessandra Polissi, alessandra.polissi@unimi.it; Waldemar Vollmer, w.vollmer@ncl.ac.uk

INDEX

**Figure S1**	Deletion of *actS* does not rescue lysis phenotype of *araB*p*lptC* Δ*ldtD* and *araB*p*lptC* Δ*ldtE* mutants.
**Figure S2**	Deletion of *actS* does not affect growth and morphology of BW25113 (*lptC*$^{+}$) cells.
**Figure S3**	Deletion of *actS* along with that of *ldts* does not affect growth and morphology of BW25113 (*lptC*$^{+}$) cells.
**Figure S4**	Ectopic expression of *actS* in *araB*p*lptC* does not induce lysis.
**Figure S5**	ActS activates AmiA and AmiB.
**Figure S6**	Deletion of *amiA, amiB amiC* or *nlpD* does not rescue the lysis phenotype of the *araB*p*lptC* Δ*ldtF* mutant.

**Table S1**	Muropeptide composition of *araB*p*lptC, araB*p*lptC* Δ*actS* and *araB*p*lptC* Δ*ldtF Δ actS* mutants under permissive or nonpermissive conditions (separate file).
**Table S2**	Bacterial Strains used in this study.
**Table S3**	Plasmids used in this study.
**Table S4**	Oligonucleotides used in this study.

Figure S1

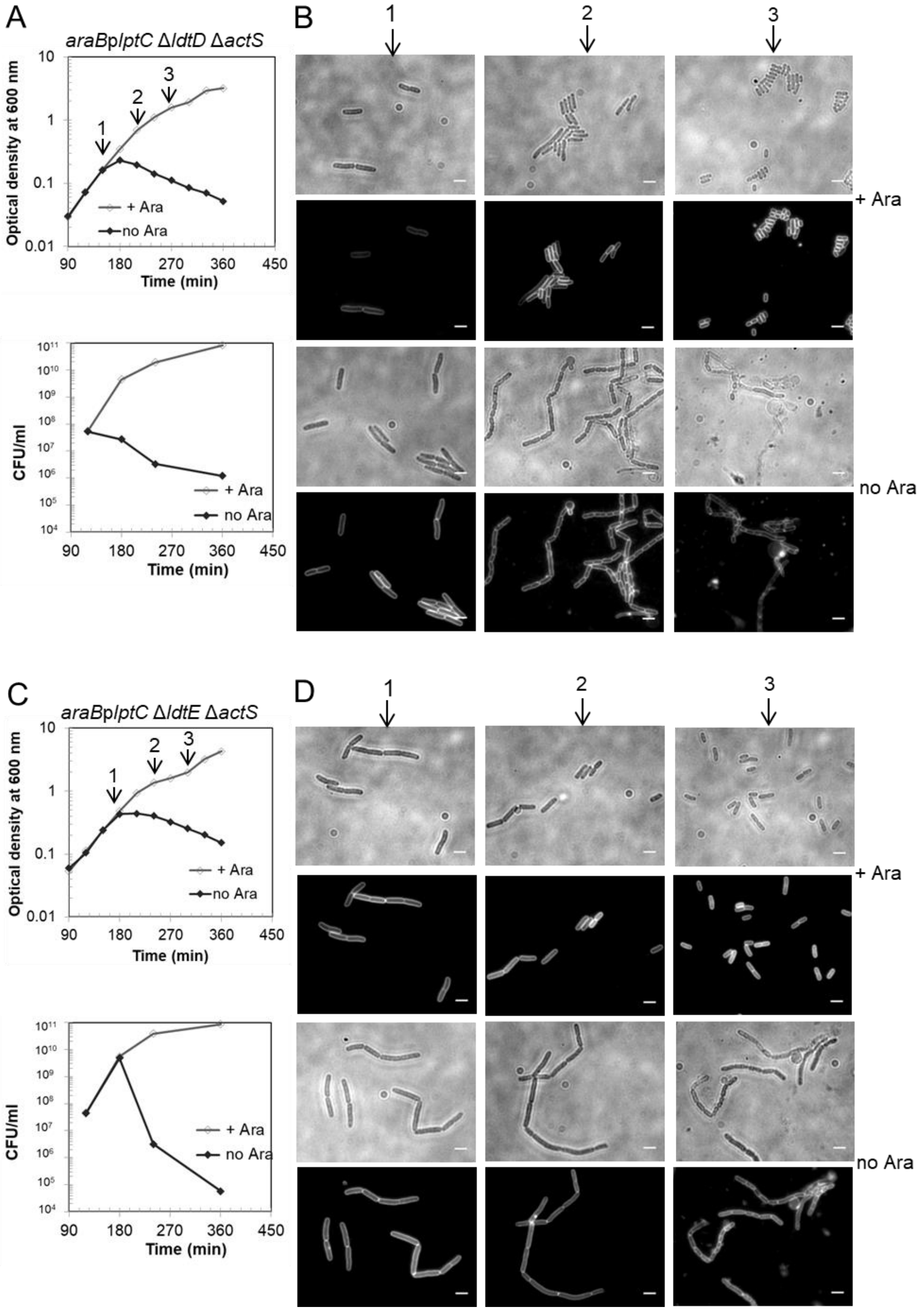

Figure S1. Deletion of *actS* does not rescue lysis phenotype of *araB*p*lptC* Δ*ldtD* and *araB*p*lptC* Δ*ldtE* mutants. The strains *araB*p*lptC* Δ*ldtD* Δ*actS* (**A**) and *araB*p*lptC* Δ*ldtE* Δ*actS* (**C**) were grown in the presence of 0.2% arabinose to an OD_{600} of 0.2, harvested, washed three times, and resuspended in an arabinose-supplemented (+ Ara) or arabinose-free (no Ara) medium. Growth was monitored by OD_{600} measurements (top panel) and by determining CFU (bottom panel). Growth curves are representative of at least three independent experiments. At t=150, 210, and 270 min (arrows), *araB*p*lptC* Δ*ldtD* Δ*actS* (**B**) and *araB*p*lptC* Δ*ldtE* Δ*actS* (**D**) cells were imaged. Phase-contrast images (top) and fluorescence images (bottom) are shown. Bars, 3 μm.

Figure S2

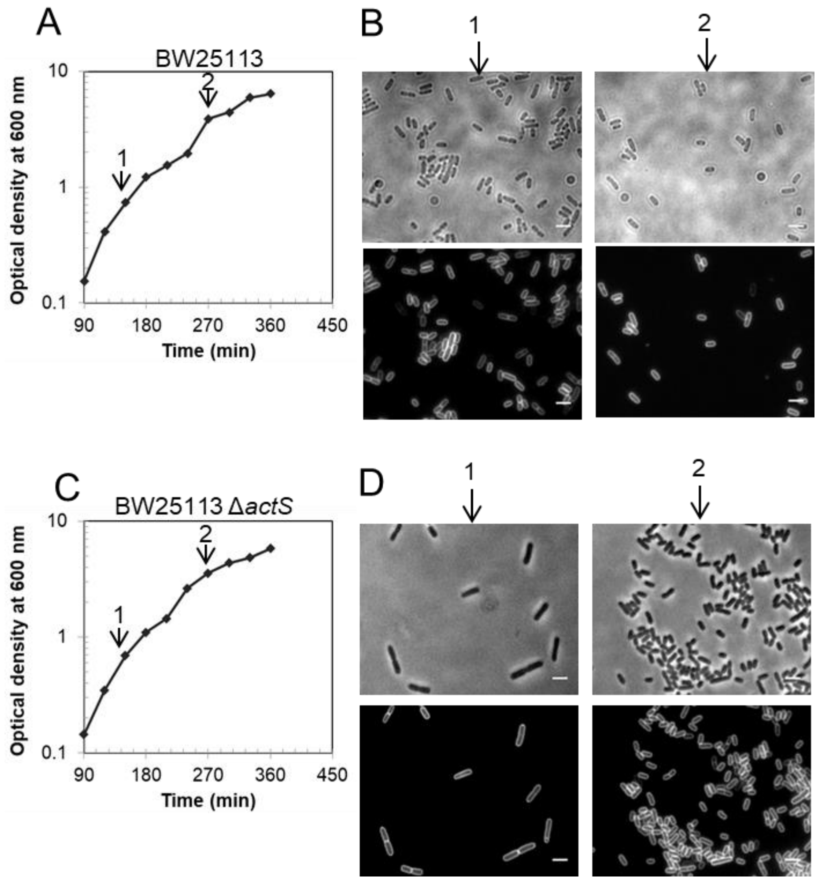

Figure S2. Deletion of *actS* does not affect growth and morphology of BW25113 (*lptC*$^{+}$) cells. Growth of BW25113 (**A**) and BW25113 Δ*actS* (**C**) was monitored by OD_{600} measurements. Growth curves are representative of at least three independent experiments. At t=120 and 270 min (arrows), BW25113 (**B**) and BW25113 Δ*actS* (**D**) cells were collected for imaging. Phase-contrast images (top) and fluorescence images (bottom) are shown. Bars, 3 μm.

Figure S3

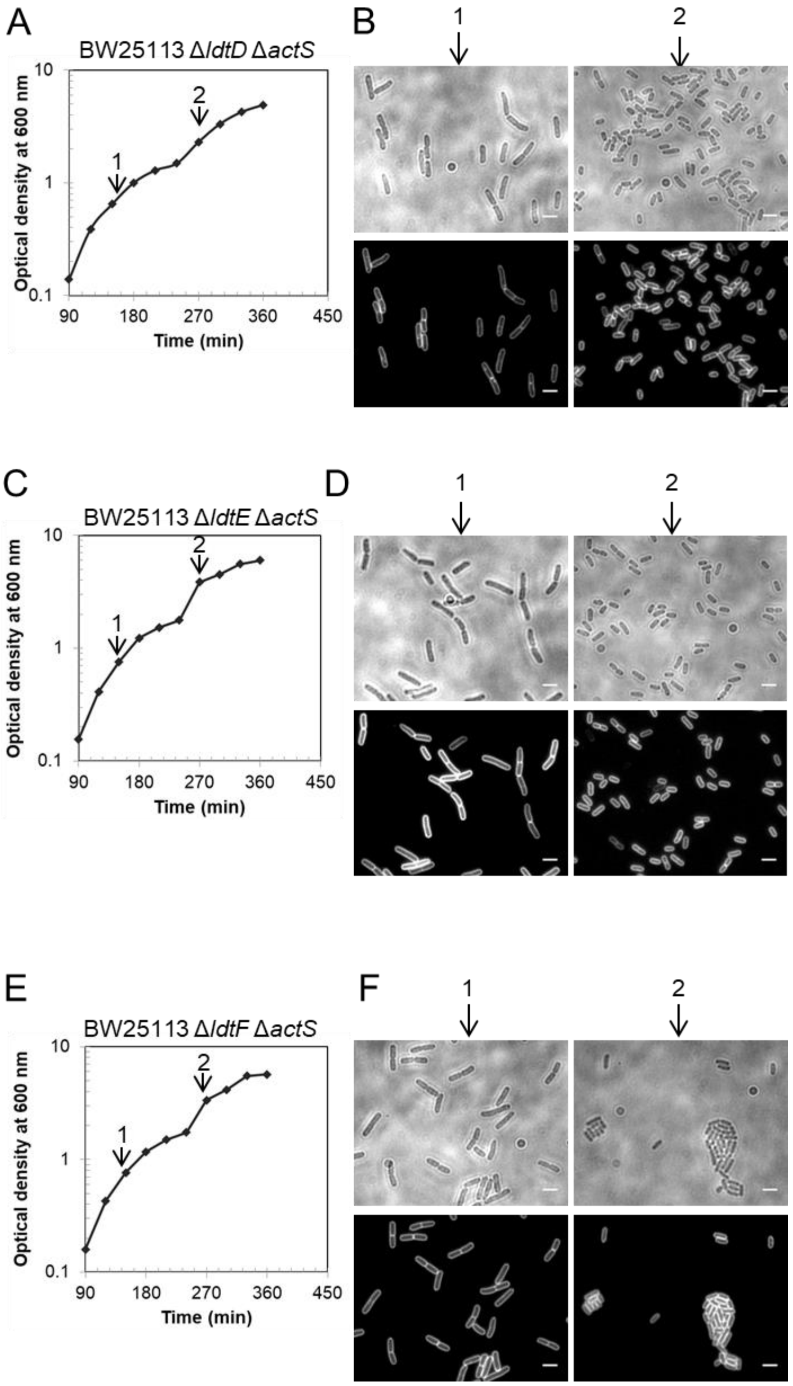

Figure S3. Deletion of *actS* along with that of *ldts* does not affect growth and morphology of BW25113 (*lptC*$^+$) cells. Growth of BW25113 Δ*ldtD* Δ*actS* (**A**), BW25113 Δ*ldtE* Δ*actS* (**C**) and BW25113 Δ*ldtF* Δ*actS* (**E**) cells was monitored by OD_{600} measurements. Growth curves are representative of at least three independent experiments. At t=120 and 270 min (arrows), BW25113 Δ*ldtD* Δ*actS* (**B**), BW25113 Δ*ldtE* Δ*actS* (**D**) and BW25113 Δ*ldtF* Δ*actS* (**F**) cells were collected for imaging. Phase-contrast images (top) and fluorescence images (bottom) are shown. Bars, 3 μm.

Figure S4

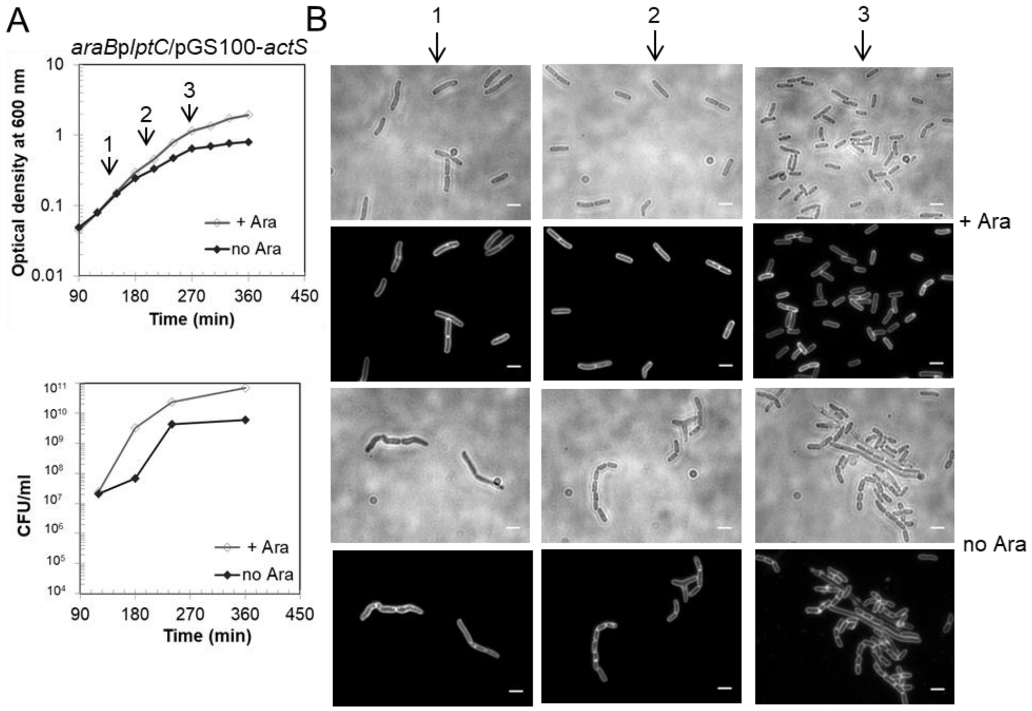

Figure S4. Ectopic expression of *actS* in *araB*p*lptC* does not induce lysis. Cells of *araB*p*lptC* harboring pGS100-*actS* were grown in the presence of 0.2% arabinose to an OD_{600} of 0.2, harvested, washed three times, and resuspended in an arabinose-supplemented (+ Ara) or arabinose-free (no Ara) medium. (**A**) Growth was monitored by OD_{600} measurements (top panel) and by determining CFU (bottom panel). Growth curves are representative of at least three independent experiments. At t=150, 210, and 270 min (arrows), samples were imaged. (**B**) Phase-contrast images (top) and fluorescence images (bottom) are shown. Bars, 3 μm.

Figure S5

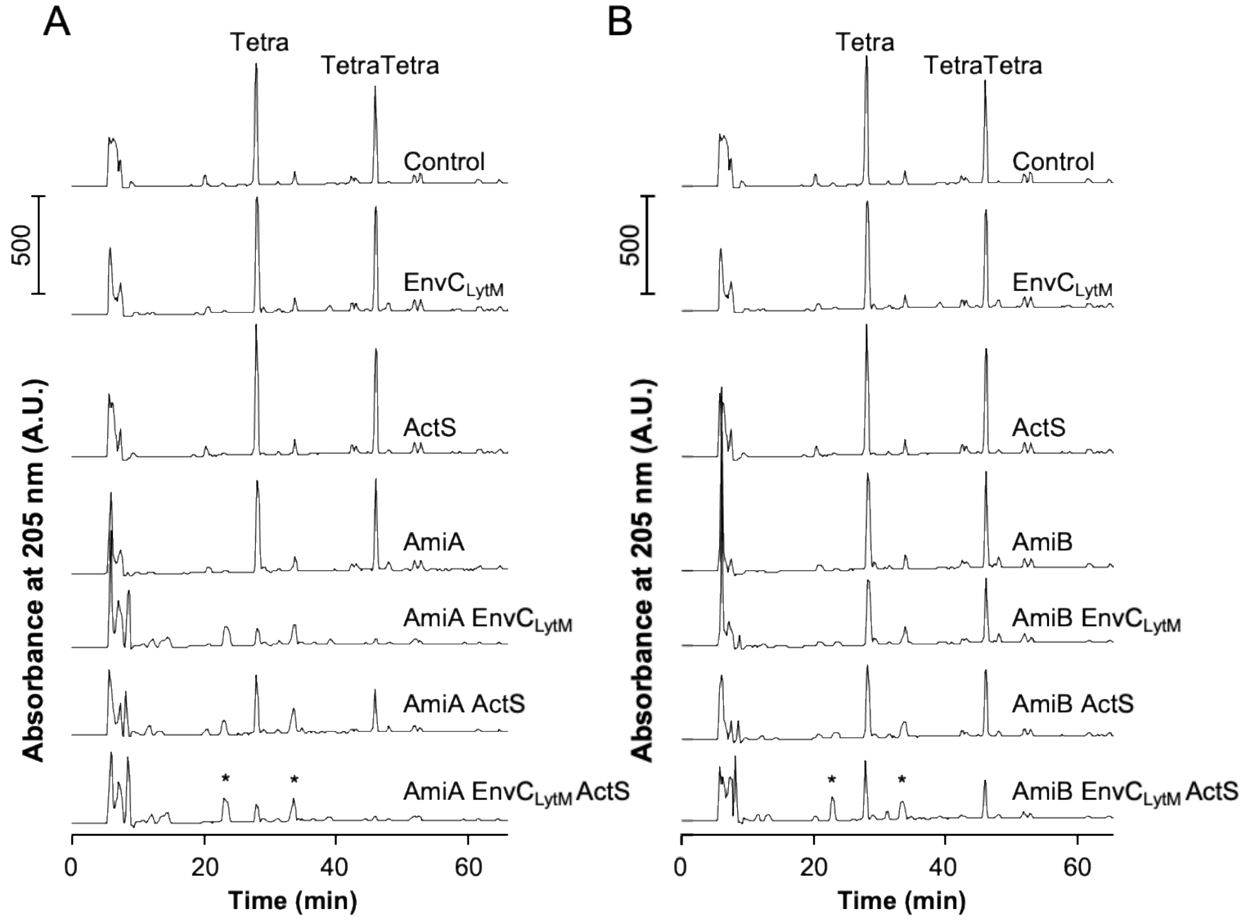

Figure S5. ActS activates AmiA and AmiB. HPLC chromatograms showing the activation of (**A**) AmiA and (**B**) AmiB by their cognate activator $EnvC_{LytM}$ and ActS. *E. coli* PG was incubated with proteins indicated on the right side. Reactions were terminated by boiling for 10 min. Samples were digested with cellosyl and the resulting muropeptides were reduced with sodium borohydride and separated by reversed-phase HPLC. Asterisks indicate products of amidase activity. The structures of Tetra and TetraTetra are shown in Figure 4.

Figure S6

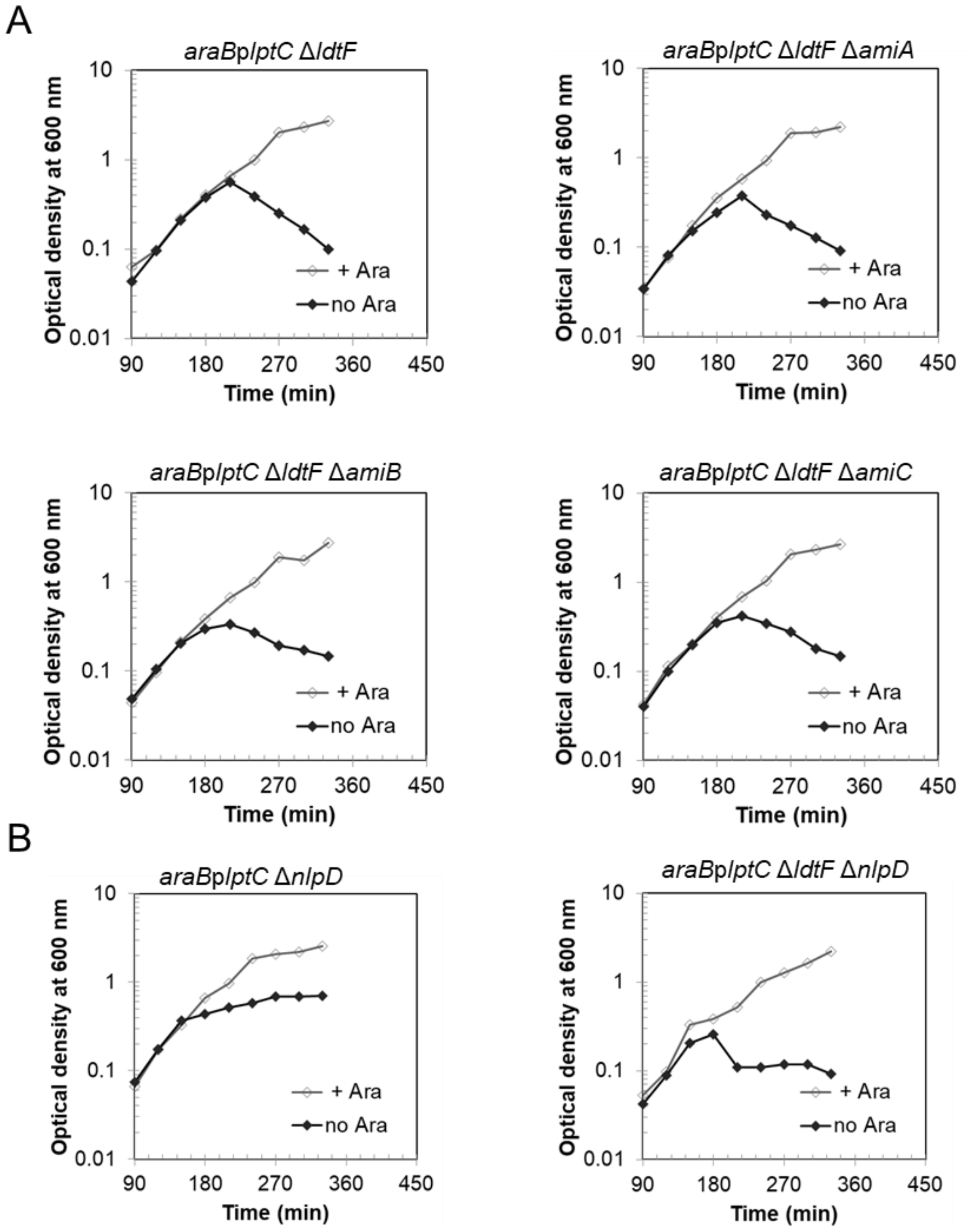

Figure S6. **Deletion of *amiA, amiB, amiC* or *nlpD* does not rescue the lysis phenotype of the *araB*p*lptC* Δ*ldtF* mutant.** Cells of *araB*p*lptC* Δ*ldtF*, *araB*p*lptC* Δ*ldtF* Δ*amiA*, *araB*p*lptC* Δ*ldtF* Δ*amiB*, *araB*p*lptC* Δ*ldtF* Δ*amiC* (A) and *araB*p*lptC* Δ*nlpD*, *araB*p*lptC* Δ*ldtF* Δ*nlpD* (B) were grown in the presence of 0.2% arabinose to an OD$_{600}$ of 0.2, harvested, washed three times, and resuspended in an arabinose-supplemented (+ Ara) or arabinose-free (no Ara) medium. Growth was monitored by OD$_{600}$ measurements. Growth curves are representative of at least three independent experiments.

Table S2. Bacterial strains.

Strain	Relevant genotype or features	Source or reference
AMM24	BW25113 Δ*ldtF*::frt	Morè *et al*., 2019
AMM30	BB-3 Δ*ldtF*::frt	Morè *et al*., 2019
AMM39	BW25113 Δ*actS*::frt	This work
AMM43	BW25113 Δ*actS*::frt Δ*ldtD*::frt	This work
AMM44	BW25113 Δ*actS*::frt Δ*ldtE*::frt	This work
AMM45	BW25113 Δ*actS*::frt Δ*ldtF*::frt	This work
AMM46	BB-3 Δ*actS*::frt	This work
AMM47	BB-3 Δ*ldtD*::frt Δ*actS*::frt	This work
AMM48	BB-3 Δ*ldtE*::frt Δ*actS*::frt	This work
AMM49	BB-3 Δ*ldtF*::frt Δ*actS*::frt	This work
AMM88	BW25113 *ΔldtF::frt ΔnlpD::frt*	This work
AMM89	BB-3 *ΔldtF::frt ΔnlpD::frt*	This work
AMM90	BB-3 *ΔnlpD::frt*	This work
BB-3	BW25113 Φ(*kan araC araB*p*lptC*)1	Sperandeo *et al*., 2006
BL21(DE3)	F– *ompT hsd*SB(rB– mB–) *gal dcm* (DE3)	Novagen
BW25113	*lacI*^{q} *rrnB*$_{T14}$ Δ*lacZ*$_{WJ16}$ *hsdR514* Δ*araBAD*$_{AH33}$ Δ*rhaBAD*$_{LD78}$	Datsenko and Wanner, 2000
BW25113Δ6LDT	*ΔycbB ΔerfK ΔycfS ΔybiS ΔynhG ΔyafK*	Kuru *et al*. 2017
CKG02	BW25113 Δ*nlpD*::frt	This work
CKG04	BW25113 Δ*envC*::frt	This work
CKG06	BW25113 Δ*amiA*::frt	This work
CKG08	BW25113 Δ*amiB*::frt	This work
CKG10	BW25113 Δ*amiC*::frt	This work
CKG12	BW25113 Δ*nlpD*::frt Δ*envC*::frt	This work
CKG14	BW25113 Δ*amiA*::frt Δ*amiC*::frt	This work
CKG16	BW25113 Δ*amiA*::frt Δ*amiB*::frt Δ*amiC*::frt	This work
CKG18	BW25113 Δ*envC*::frt Δ*amiC*::frt	This work
CKG20	BW25113 Δ*nlpD*::frt Δ*amiB*::frt	This work
CKG21	BW25113 Δ*nlpD*::frt Δ*amiA*::*kan* Δ*amiB*::frt	This work
CKG22	BB-3 Δ*ldtF*::frt Δ*amiA*::frt	This work
CKG23	BB-3 Δ*ldtF*::frt Δ*amiB*::frt	This work
CKG24	BB-3 Δ*ldtF*::frt Δ*amiC*::frt	This work
DH5α	Δ(*argF-lac169*) ϕ80 *dlacZ58*(M15) *glnV44*(AS) λ^{-} *rfbD1 gyrA96 recA1 endA1 spoT1 thi-1 hsdR17*	Hanahan, 1983
JW2428	BW25113 Δ*amiA*764::*kan*	Baba *et al*., 2006
JW2712	BW25113 Δ*nlpD*747::*kan*	Baba *et al*., 2006
JW2833	BW25113 Δ*ygeR*787::*kan*	Baba *et al*., 2006
JW4127	BW25113 Δ*amiB*790::*kan*	Baba *et al*., 2006
JW5449	BW25113 Δ*amiC*742::*kan*	Baba *et al*., 2006
JW5646	BW25113 Δ*envC*725::*kan*	Baba *et al*., 2006
LOBSTR-BL21(DE3)	F– *ompT hsd*SB(rB– mB–) *gal dcm* (DE3), carries genomically modified copies of *arnA* and *slyD*	Kerafast
MC1061	*araD*139 Δ(*araA*-leu)7697 Δ(*lac*)X74 *galK*16 *galE*15(GalS) lambda- e14- *mcrA0 relA*1 *rpsL*150(*str*R) *spoT*1 *mcrB*1 *hsdR*2	Casadaban and Cohen, 1980

Table S3. Plasmids.

Plasmids	Relevant characteristics	Source or reference
pCP20	FLP expression, temperature sensitive replication; Cam^R and Amp^R.	Datsenko and Wanner, 2000
pET28a His-*actS*	pET28a derivative; expresses *actS* from the T7 promoter starting from amino acid 27 and fused at N-terminal with 6xHis tag.	This work
pGS100	pGZ119EH derivative, contains TIR sequence downstream of p*tac*, Cam^R.	Sperandeo *et al*., 2006
pGS100-*actS*	pGZ119H derivative; expresses full length *actS*$^{(1-251)}$ under the *tac* promoter; Cam^R.	This work
pGS100-*actS*$_{LysM}$	pGZ119H derivative; expresses *a actS* construct containing the signal sequence and the LysM domain (1-106 residues) under the *tac* promoter; Cam^R.	This work
pGS100-*actS*$_{LytM}$	pGZ119H derivative; expresses a chimeric version of *actS* containing the signal sequence (1-39 residues) fused to the LytM domain (84-251 residues) under the *tac* promoter; Cam^R.	This work
pKD46	λ-Red expression under the *araB*p promoter, temperature sensitive replication; Amp^R.	Datsenko and Wanner, 2000
pET28a-His-EnvC	pET28a derivative; expresses *envC* from the T7 and fused at N-terminal with 6xHis tag.	This work
pTU104	bla lacIq PT7::h-sumo-lytenvC	Uehara *et al.*, 2010
pTU119	bla lacIq PT7::h-sumo-nlpD(27-379)	Uehara *et al*., 2010
pTB327	bla lacIq PT7::h-sumo-amiA(34-289)	Uehara *et al.*, 2010
pTB324	bla lacIq PT7::h-sumo-amiB(23-445)	Uehara *et al*., 2010
pTU203	bla lacIq PT7::h-sumo-amiC(32-418)	Uehara *et al*., 2010

Table S4. Oligonucleotides.

Name	Sequence (5'-3')	Description	Used to make
AP618/30 *actS*-f	CAGGAATTCAACTTGAGTGCGGGACGCCTG	[CAG]-[EcoRI]-[AAC]-[start *actS*; fwd]	pGS100-*actS*<br>pGS100-$actS_{\mathrm{LysM}}$<br>pGS100-$actS_{\mathrm{LytM}}$
AP619/29 *actS*-r	TATCAAGCTTTCAGCATTTTGGCTTGCTG	[TATC]-[HindIII]-[stop *actS*; rev]	pGS100-*actS*<br>pGS100-$actS_{\mathrm{LysM}}$<br>pGS100-$actS_{\mathrm{LytM}}$
AP621/ 33 *actS*-r	CCGCTCGAGTCAGCATTTTGGCTTGCTGCCCTG	[CCG]-[XhoI]-[stop *actS*; rev]	pET28a-His-ActS
AP677 28 *actS*26-f	AGCCATATGTCGGGTAGCAAATCATCCG	[AGC]-[NdeI]-[starting at 78 bp downstream of TTG of *actS*; fwd]	pET28a-His-ActS
AP703/33 *actS*int-r	TATCAAGCTTTCATGCGGTTTTGGTCGTTGATT	[TATC]-[HindIII]-[TC]-[316 bp downstream of TTG of *actS*; rev]	pGS100-$actS_{\mathrm{LysM}}$
AP704/40 *actS*ssLytM-r	CTGCTACTTTTCGCCCCACCGCCGGAATACGTTCCTGTAT	[117 bp downstream of TTG of actS]-[252 bp downstream of TTG of *actS*; rev]	pGS100-$actS_{\mathrm{LytM}}$
AP705/ 20 *actS*LytM-f	GGTGGGGCGAAAAGTAGCAG	[252 bp downstream of TTG of actS; rev]	pGS100-$actS_{\mathrm{LytM}}$
envC-f	GGAATTCCATATGGATGAGCGTGACCAACTC	[GGAATTC]-[NdeI]-[starting at 113bp downstream of ATG of *envC*; fwd]	pET28a-His-EnvC
envC-r	CCCAAGCTTTTATCTTCCCAACCACGGC	[CCC]-[HindIII]-[stop *envC;* rev]	pET28a-His-EnvC

Supplemental References

Baba, T., Ara, T., Hasegawa, M., Takai, Y., Okumura, Y., Baba, M., *et al.* (2006) Construction of *Escherichia coli* K-12 in-frame, single-gene knockout mutants: the Keio collection. *Mol Syst Biol* **2**: 2006.0008.

Casadaban, M.J., and Cohen, S.N. (1980) Analysis of gene control signals by DNA fusion and cloning in *Escherichia coli*. *J Mol Biol* **138**: 179–207.

Datsenko, K.A., and Wanner, B.L. (2000) One-step inactivation of chromosomal genes in *Escherichia coli* K-12 using PCR products. *PNAS* **97**: 6640–6645.

Hanahan, D. (1983) Studies on transformation of *Escherichia coli* with plasmids. *J Mol Biol* **166**: 557–580.

Kuru, E., Lambert, C., Rittichier, J., Till, R., Ducret, A., Derouaux, A., et al. (2017) Fluorescent D-amino-acids reveal bi-cellular cell wall modifications important for *Bdellovibrio bacteriovorus* predation. *Nat Microbiol* **2**: 1648–1657.

Morè, N., Martorana, A.M., Biboy, J., Otten, C., Winkle, M., Serrano, C.K.G., *et al.* (2019) Peptidoglycan remodeling enables *Escherichia coli* to survive severe outer membrane assembly defect. *mBio* **10**: e02729-18.

Sperandeo, P., Pozzi, C., Dehò, G., and Polissi, A. (2006) Non-essential KDO biosynthesis and new essential cell envelope biogenesis genes in the *Escherichia coli yrbG-yhbG* locus. *Res Microbiol* **157**: 547–558.

Uehara, T., Parzych, K.R., Dinh, T., and Bernhardt, T.G. (2010) Daughter cell separation is controlled by cytokinetic ring-activated cell wall hydrolysis. *EMBO J* **29**: 1412–1422.
